# Social and Emotional Values of Sounds Influence Human (*Homo sapiens*) and Non-Human Primate (*Cercopithecus campbelli*) Auditory Laterality

**DOI:** 10.1371/journal.pone.0006295

**Published:** 2009-07-17

**Authors:** Muriel Basile, Alban Lemasson, Catherine Blois-Heulin

**Affiliations:** Université Rennes 1, CNRS, UMR 6552 Ethologie Animale et Humaine, Paimpont, France; L'université Pierre et Marie Curie, France

## Abstract

The last decades evidenced auditory laterality in vertebrates, offering new important insights for the understanding of the origin of human language. Factors such as the social (e.g. specificity, familiarity) and emotional value of sounds have been proved to influence hemispheric specialization. However, little is known about the crossed effect of these two factors in animals. In addition, human-animal comparative studies, using the same methodology, are rare. In our study, we adapted the head turn paradigm, a widely used non invasive method, on 8–9-year-old schoolgirls and on adult female Campbell's monkeys, by focusing on head and/or eye orientations in response to sound playbacks. We broadcast communicative signals (monkeys: calls, humans: speech) emitted by familiar individuals presenting distinct degrees of social value (female monkeys: conspecific group members *vs* heterospecific neighbours, human girls: from the same *vs* different classroom) and emotional value (monkeys: contact *vs* threat calls; humans: friendly *vs* aggressive intonation). We evidenced a crossed-categorical effect of social and emotional values in both species since only “negative” voices from same class/group members elicited a significant auditory laterality (Wilcoxon tests: monkeys, T = 0 p = 0.03; girls: T = 4.5 p = 0.03). Moreover, we found differences between species as a left and right hemisphere preference was found respectively in humans and monkeys. Furthermore while monkeys almost exclusively responded by turning their head, girls sometimes also just moved their eyes. This study supports theories defending differential roles played by the two hemispheres in primates' auditory laterality and evidenced that more systematic species comparisons are needed before raising evolutionary scenario. Moreover, the choice of sound stimuli and behavioural measures in such studies should be the focus of careful attention.

## Introduction

Since the end of the nineteenth century, auditory laterality, representing a functional specialization of only one brain hemisphere to process sounds, was believed to be a human specificity. This asymmetric hemispheric implication, early evidenced by clinical observations on human aphasic patients (*Homo sapiens*) for the processing of linguistic features [1], [2] rather corresponds to a task sharing between the two hemispheres than to a dominance of one hemisphere over the other [3], [4]. The left hemisphere would be specialized for the processing of syntactic and semantic activities, whereas the right hemisphere could attend preferentially to the prosody or novelty of a signal [5]–[8]. The last decades evidenced laterality in vertebrates and thus raised important insights for the understanding of the origin of human language [9]–[11]. Nevertheless, comparative studies between humans and animals are still needed. While sharing general anatomical and genetic similarities with humans, non-human primates also exhibit notable similarities concerning the nervous circuitry processing vocal production and auditory perception [12], [13]. For these reasons, monkeys would constitute an ideal candidate for such a comparison.

Some authors showed that auditory laterality was influenced by the social value of the sound processed. Studies first tested the influence of a sound's species-specificity. The perception of conspecific calls highlighted a left (Japanese macaque (*Macaca fuscata*): [12], [14], [15]; rhesus macaque (*Macaca mulatta*): [3], [16]; California sea lion (*Zalophus californianus*): [17]; dog (*Canis lupus*): [18]) or right hemispheric preference (starling (*Sturnus vulgaris*): [19], [20]; chimpanzee (*Pan troglodytes*): [21]) depending on the species studied. Several authors found that familiarity with the sound heard influenced the hemispheric specialisation in some species (zebra finche (*Taeniopygia guttata*): [22]; starling (*S. vulgaris*): [19], [20]; bonobo (*Pan paniscus*): [23]; horse (*Equus caballus*): [24]), but not in others (California sea lion (*Z. californianus*): [17]; vervet monkey (*Chlorocebus aethiops*): [25]). Finally, George et al. [20] evidenced in starlings that neural lateralisation could differ for songs expressing distinct social functions (short *vs* long distance communication). These non-consensual results suggest that more systematic investigations comparing humans and animals and based on similar approaches, paradigms and analyses are needed to understand the influence of a sound's social value.

In parallel, hemispheric specialization can also be influenced by the emotional value of the signal. An asymmetrical processing of emotion was largely assessed by the literature in humans (*Homo sapiens*), leading to the establishment of two dominant theories: “the Right Hemisphere theory” and “the Valence theory”. The Right Hemisphere theory defends a predominance of the right hemisphere for the processing of any stimuli expressing high emotional value [26], [27]. The valence theory defends rather a differential implication of the left and the right hemisphere to process stimuli depending on their emotional valence. Thus, positive emotional stimuli would be preferentially processed by the left hemisphere, while the right hemisphere would be specialized for the processing of negative emotions. In humans, right hemisphere predominance for emotional linguistic auditory stimuli was assessed in stroke patients [28], as well as for the processing of emotional intonations in normal subjects [29]. Moreover, the right hemisphere treats preferentially emotive features, whereas the left hemisphere is more sensitive to the lexical semantic content of emotional prosodic stimuli in speech [4]. To our knowledge, the influence of emotion on non-human primates' laterality has up to now principally been tested through the visual modality (Gelada baboon (*Theropithecus gelada*): [30]; rhesus macaque (*M. mulatta*): [31]; chimpanzee (*P. troglodytes*): [32]. One study investigated the effect of emotion on laterality through the auditory modality in non-human primates [33]. This work showed a sex dependent right ear/left hemisphere bias in male grey mouse lemurs (*Microcebus murinus*) for the processing of species-specific calls with a negative emotional value, while calls with a positive value elicited no such asymmetry. However, it did not validate any of the two theories. We therefore found particularly interesting to investigate the crossed effect of the social and emotional values of communicative sounds on auditory laterality comparatively in humans and monkeys.

Several authors have successfully used the head-turn paradigm as a non-invasive way to assess auditory laterality [16], [17], [23], [33]–[37]. The head orientation to one side, in reaction to a stimulus broadcast directly behind the subject, would be an indicator of a privileged use of one ear resulting in a crossed processing of the auditory information by the controlateral hemisphere [33], [38]. Streri [39] emphasized in human newborns a relation between auditory lateralization (measured electrophysiologically) and gaze orientations (behavioural observation) (see Table 1 for a review). We thus adapted the head-turn paradigm, by focusing on head orientations and gaze orientations, in response to a sound broadcast at 180° behind the subject. We used these two behavioural reactions as visible clues for the ear preference to investigate auditory laterality.

**Table 1:** Example of demonstrations of auditory discrimination or laterality, involving gaze and/or head orientation measures.

Authors	Subjects	Tested variables	Types of tests	Material processed	Results
[40]	Newborns	Head orientation	Behavioural observations	None	Newborns show spontaneous directional bias for head orientations 12 hours after birth
[41]	Newborns	Gaze orientation	Monaural tests	Sound	Newborns orient their gaze in the direction of a sound presented laterally
[41]	Newborns	Gaze orientation	Bilateral tests	Speech	Newborns orient their gaze to the right when hearing a linguistic sound presented bilaterally (LH)
[41]	Newborns	Head orientation	Bilateral tests	Sound	Newborns orient their head to the left when hearing a white noise simultaneously to a linguistic sound (RH)
[42]	Newborns	Head orientation	Bilateral tests	Speech	Newborns orient their head to the right when hearing a linguistic sound presented bilaterally (LH)
[43]	Adults	Gaze orientation	Dichotic tests	Music	An eccentric gaze modifies sensitivity to sound localisation and recognition
					Lateral eye movements help to localize musical sound
[44]	Adults	Gaze orientation	Dichotic tests	Speech	Adults express consistency for the right ear preference when instructions to orient head or gaze are given (LH)
		Head orientation			Adults express an even stronger right ear preference when the decision to orient head or gaze was made by the subject (LH)
[45]	Adults	Gaze orientation	Dichotic tests	Sound	An eccentric gaze modifies sensitivity to sound lateralization
					The gaze direction influences the direction of the auditory lateralization

LH: left hemisphere processing/RH: right hemisphere processing.

Human auditory laterality for emotional speech has largely been investigated in the last decade, but far less in human infants. However, even newborns exhibit auditory laterality and show abilities for social and emotional discrimination (Table 2). Infants develop sensitivity to the mother's voice familiarity, progressively extract linguistic and emotional content from speech and succeed in identifying and labelling emotions, with higher scores for girls from the age of four. Moreover, from the age of five children exhibit a left hemispheric specialisation for verbal components in speech and a right hemispheric specialisation for the processing of emotional content. Finally, Berndt & Hoyle [54], by analysing the stability of social affinities at school on 7–10-year- old children, evidenced a consistency in friendship during six consecutive months.

**Table 2:** Example of demonstrations of emotional discrimination and auditory laterality in human infants of various ages.

Thematic	Authors	Subjects	Types of tests	Tested variables	Material processed	Objectives	Results
*Social discrimination*	[46]	Newborns (4 months old)	Behavioural observations	Time spent looking at the stimuli	Movies (vocal & facial expressions)	Discrimination of familiarity (maternal links)	Effective discrimination of mother voice for affective stimuli
*Emotional discrimination*	[47]	Newborns (5 months old)	Behavioural observations	Time spent looking at the stimuli & associated behaviours	Pictures (vocal & facial expressions)	Discrimination of emotional expressions	Effective discrimination and correct labelling of emotional expressions
	[48]	Children (3–5 years old)	Several	Emotional labelling : happiness & angriness	- Video channel alone	Identification and labelling of emotions through different channels of presentation	Expression of the same ability than adults to identify and label correctly emotions regardless of the channel of presentation
					- Audio channel alone		
					- Audio-video channel		
	[49]	Children (3–9 years old)	Binaural tests	Emotional labelling : happiness, angriness & neutrality	Speech (Words)	Identification and labelling of emotions	Increase of the reliability to correctly recognize positive and negative emotions with age
						Reliability with age	Less sensitivity to the neutral components in children
	[50]	Children (4 years old)	Interviews/Standard vocabulary tasks	Emotional labelling : happiness, sadness, pride & embarrassment	Puppets	Identification and labelling of emotions	Greater ability of girls to label and understand emotions compared to boys
					Self and peer- explanations		Effective recognition of happiness and sadness
*Auditory laterality*	[51]	Newborns (0–1 day old)	Evoked potentials/Bilateral tests	Cerebral activation	Speech	Lateralisation of speech	Higher cerebral activation in the left hemisphere (LH)
	[52]	Children (2–5 years old)	Dichotic tests	Ear preference	Speech (Digits)	Reliability with time (test/re-test)	Expression of a right ear preference (LH) for 2/3rds of the subjects
	[53]	Children (5–14 years old)	Dichotic tests	Ear preference	Speech (verbal and emotional content)	Lateralisation of speech	Expression of a right ear preference (LH) to report verbal content
							Expression of a left ear preference (RH) to report emotional content

*LH: left hemisphere processing/RH: right hemisphere processing*.

Campbell's monkeys (*Cercopithecus campbelli*) appeared as a good non-human primate candidate for a comparison. This species has recently been studied intensively. Authors revealed rare and complex abilities in their vocal communicative abilities (semantic, syntax: [55], [56], Ouattara et al., revised). Moreover, a socially-influenced acoustic plasticity has been found in females' contact calls [57]. Adult females perform vocal sharing by producing calls with similar frequency contours to those of their preferred partners [58], [59].

In this study, we compared, using the exact same protocol, the auditory laterality of 8–9-year-old human girls with adult female Campbell's monkeys, for the processing of sounds differing in their social and/or emotional values. Two different categories of social values were selected, e.g. intra- and inter- social group emitters (i.e. humans: girl mates from the same classroom *vs* same age familiar girls from another classroom, monkeys: conspecific adult females from the same harem group *vs* familiar heterospecific adult females living in a neighbouring enclosure). Within these social categories, two different sub-categories of emotional values were tested, e.g. positive and negative sounds (e.g. humans: sentence pronounced with a friendly *vs* aggressive intonation, monkeys: contact *vs* threat calls). We then explored through the type of behavioural responses observed (gaze or head orientation) whether laterality differed according to the category of stimuli diffused.

## Results

### Reactivity to stimuli

#### Gaze orientation

The monkeys responded extremely rarely only by moving their eyes (GO) (0%–25% - Table 3). The number of non-responses by gaze orientation was significantly higher than the number of responses, whatever the session (Wilcoxon tests, n = 7, T =  0 p = 0.02). The girls oriented more frequently their gaze (38–52%) but the differences between the number of responses and non-responses were not significant (Wilcoxon tests, n = 13, 6.5<T<25.5, p>0.05 - Table 4). Moreover, in both species, the highest percentages of response were obtained after the diffusion of intra-group sounds regardless of emotion (IGP – IGN, monkeys: 12%/girls: 52%). Subjects individually exhibited percentages of reactivity ranging from 2% to 17% in monkeys and from 19% to 75% in girls (Binomial tests, p<0.05).

**Table 3:** Percentages of reactivity expressed for gaze orientations, head orientations and First Reactions by monkeys for each sound category.

Stimuli	Session	Gaze orientation	Head orientation	First reaction
Intra-group positive	S1	18 ns	100 *	100 *
	S2	7 ns	100 *	100 *
	STOT	12 ns	100 *	100 *
Intra-group negative	S1	25 ns	96 *	100 *
	S2	0 ns	100 *	100 *
	STOT	12 ns	98 *	100 *
Extra-group 1 positive	S1	18 ns	96 *	96 *
	S2	0 ns	100 *	100 *
	STOT	9 ns	98 *	98 *
Extra-group 1 negative	S1	7 ns	100 *	100 *
	S2	0 ns	96 *	96 *
	STOT	4 ns	98 *	98 *
Extra-group 2 positive	S1	4 ns	100 *	100 *
	S2	9 ns	100 *	100 *
	STOT	9 ns	100 *	100 *
Extra-group 2 negative	S1	11 ns	100 *	100 *
	S2	9 ns	89 *	89 *
	STOT	9 ns	95 *	95 *
Control sound	S1	7 ns	93 *	93 *
	S2	7 ns	86 *	86 *
	STOT	7 ns	89 *	89 *
TOTAL	S1	13 ns	99 *	99 *
	S2	5 ns	96 *	97 *
	STOT	9 ns	97 *	98 *

Asterisk result of Wilcoxon test: *: p<0.05, ns: non significant.

**Table 4:** Percentages of reactivity expressed for gaze orientations, head orientations and First Reactions by schoolgirls for each sound category.

Stimuli	Gaze orientation	Head orientation	First reaction
Intra-group positive	52 ns	56 ns	90 **
Intra-group negative	52 ns	60 ns	85 **
Extra-group positive	38 ns	54 ns	79 *
Extra-group negative	50 ns	63 *	85 **
TOTAL	48 ns	58 *	85 **

Asterisk result of Wilcoxon test: **: p<0.01, *: p<0.05, ns: non significant.

#### Head orientation

The monkeys responded frequently by head orientation (HO) whatever the session (86%–100% - Table 3). Overall, the number of HO responses was significantly higher than the number of non-responses (Wilcoxon tests, n = 7, T = 0 p = 0.02), as well as for each single stimulus category taken separately (Wilcoxon tests, n = 7, T = 0 p<0.05). The control sound always triggered the lowest percentages of responses (CS, Wilcoxon tests, n = 7, T = 0 p<0.05). The girls also responded significantly frequently to the playbacks by head orientation (HO) (54%–63% - Table 4). Overall, the number of HO responses was significantly higher than the number of non-responses (Wilcoxon test, n = 13, T = 1.5 p = 0.01). However, a more thorough analysis showed that the number of responses did not significantly differ from the number of non-response in any single stimulus category (Wilcoxon tests, n = 13, 3<T<12, 0.06<p<0.34), except for extra-group negative stimuli (EGN, Wilcoxon test, n = 13, T = 0 p = 0.02). Percentages of head orientation ranged from 94% to 100% for all the monkey subjects taken individually (Binomial tests, p<0.001), bearing very few inter-individual differences. Conversely, a high inter-individual variability was observed in girls, with individual percentages of head orientation ranging from 19% (Binomial test, p<0.05) to 94% (Binomial test, p = 0.0005).

#### First response

In monkeys, when considering the first response (FR), either head or gaze orientation, a high reactivity was observed whatever the session (86%–100% - Table 3). Overall, the number of responses was significantly higher than the number of non-responses (Wilcoxon tests, n = 7, T = 0 p = 0.02), with similar or higher reactivity scores than for HO responses. When considering both head and gaze orientation (FR), schoolgirls showed the highest percentage of reactivity (79%–90% - Table 4). Overall, the number of FR responses was significantly higher than the number of non-responses (Wilcoxon test, n = 13, T = 0 p = 0.005), as well as for each single stimulus category taken separately (Wilcoxon tests, n = 13, 0<T<4, 0.01<p<0.03). Little inter-individual variability was observed both in monkeys and girls, with percentages of reactivity ranging respectively from 96% to 100% and from 50% to 100% (Binomial tests, p<0.001).

### Laterality

#### Gaze orientation

The monkeys showed more gaze orientations to the right when hearing biological sounds (all biological sounds pooled together – Wilcoxon test, n = 7, T = 1 p = 0.05). But, low levels of reactivity did not allow us to take the analysis any further. Overall, the girls showed a right side preference for gaze orientations (Wilcoxon test, n = 13, T = 4 p = 0.006). When analysing each single stimulus category separately, this remained significant only for negative extra-group stimuli (EGN, Wilcoxon test, n = 13, T = 3.5 p = 0.02).

#### Head orientation

**Figure 1:**
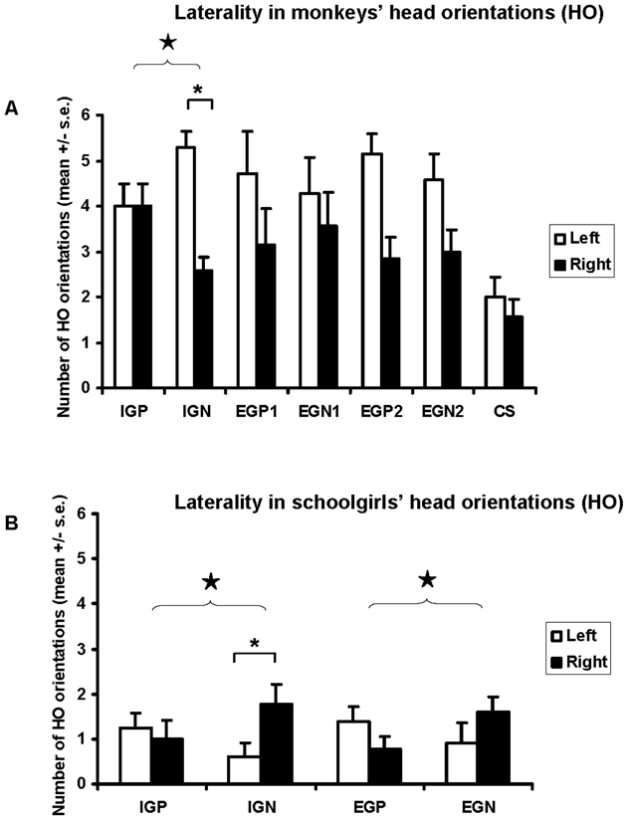
Laterality of head orientations in relation to sound categories for monkeys and girls. Values shown are the means±s.e. Stimuli – IG: intra-group, EG: extra-group, P: positive, N: negative, 1: De Brazza monkeys, 2: red-capped mangabeys, CS: control sound. Asterisk: Result of Wilcoxon test: p<0.05 Open star: Result of Fisher test: p<0.05.

Overall no side preference was observed for head orientation in the two species (Wilcoxon tests, monkeys STOT n = 7, T = 4 p = 0.09/girls n = 13, T = 16 p = 0.24). Interestingly, Campbell's monkeys showed a left head preference (suggesting a Right Hemisphere processing) when hearing intra-group negative sounds, i.e. Campbell's monkey threat calls (IGN, Wilcoxon test, n = 7, T = 0 p = 0.03 - Fig. 1A), whereas girls showed an opposite side preference (Left Hemisphere processing) for the same stimulus category, i.e. sentence pronounced by classroom mates with an aggressive intonation (IGN, Wilcoxon test, n = 13, T = 4.5 p = 0.03 - Fig. 1B). Subjects of the two species did not show any side preference when responding to any other single sound category (Wilcoxon tests; monkeys STOT n = 7, 0<T<14, 0.07<p<1/girls n = 13, 8<T<15, 0.26<p<0.67). Overall no significant side preference was observed when analysing separately the two monkey test sessions (Wilcoxon test, n = 7, S1 T = 5 p = 0.13; S2, T = 4.5 p = 0.11). Although the monkeys did not show any side preference for any single stimulus category in the first session (Wilcoxon tests, n = 7, 0<T<7.5, 0.07<p<1), they responded significantly by preferentially turning their head to the left side during the second session when hearing mangabey threat calls (EG2N, Wilcoxon test, n = 7, T = 0 p = 0.04).

We found an opposite pattern of lateralization for monkeys responding to intra-group stimuli between the processing of positive and negative sounds (Fisher tests: IGP *vs* IGN, p = 0.05; EG1P *vs* EG1N/EG2P *vs* EG2N, p>0.05 – Fig. 1A). In the case of the girls this was true for both intra- and inter-group stimuli (Fisher tests: IGN *vs* IGP, p = 0.01; EGP *vs* EGN, p = 0.03 – Fig. 1B). Thus, while more orientations to the right for negative stimuli (N) *vs* more orientations to the left for positive stimuli (P) was found for both IG and EG in girls, the monkeys displayed a left preference for negative (N) sounds *vs* no preference for positive (P) stimuli for IG only.

When comparing, within each emotional category, responses to stimuli differing in social value, no significant differences concerning the laterality patterns were found for monkeys and girls alike (Fisher tests, monkeys: IGP *vs* EG1P/IGP *vs* EG2P/EG1P *vs* EG2P/IGN *vs* EG1N/IGN *vs* EG2N/EG1N *vs* EG2N; p>0.11; girls : IGP *vs* EGP/IGN *vs* EGN; p>0.26)

#### First response

**Figure 2:**
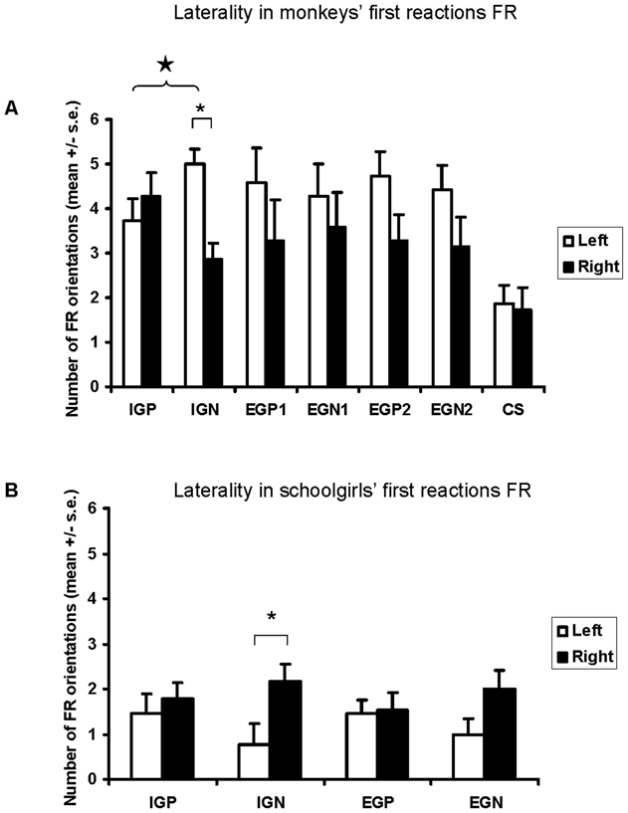
Laterality of first reactions in relation to sound categories for monkeys and girls. Values shown are the means±s.e. Stimuli – IG: intra-group, EG: extra-group, P: positive, N: negative, 1: De Brazza monkeys, 2: red-capped mangabeys, CS: control sound. Asterisk: Result of Wilcoxon test: p<0.05 Open star: Result of Fisher test: p<0.05.

Overall the monkeys and schoolgirls showed no side preference (Wilcoxon tests, monkey: n = 7, T = 3 p = 0.12; girl: n = 13, T = 5.5 p = 0.08 - Fig. 2A). For Campbell's monkey subjects, only conspecific threat calls (IGN) induced a left ear preference (Wilcoxon test, n = 7, T = 0 p = 0.04) when we considered the two sessions combined. No such preference was observed for any other single stimulus category whatever the session (S1/S2/STOT, Wilcoxon tests, n = 7, 1.5<T<8.5, 0.11<p<0.89). The schoolgirl subjects showed a right side preference (left hemisphere processing) in response to negative stimuli pronounced by a classroom mate (IGN, Wilcoxon test, n = 13, T = 6 p = 0.05), while no such preference was observed for any other single category (Wilcoxon tests, n = 13, 5<T<16, 0.44<p<0.50 – Fig. 2B).

Campbell's monkeys showed an opposite pattern of lateralization between conspecific stimuli differing in their emotional value (IGP *vs* IGN, Fisher tests, p = 0.05), whereas heterospecific calls characterized by an opposite emotional value did not show the same tendency (EG1P *vs* EG1N/EG2P *vs* EG2N, Fisher tests, p>0.41 - Fig. 2A). This particularity associated to conspecific calls (IG) showing up when the two sessions were combined disappeared when considering the two sessions separately (Fisher tests, p>0.05). Schoolgirls did not show any opposite pattern of lateralization whatever the social value considered, when we compared responses to negative stimuli *vs* positive stimuli (Fisher tests, p>0.15 - Fig. 2B).

For both species, comparison of stimuli expressing a single emotional value, but differing on the social value revealed no significant difference or opposite pattern of lateralization, whatever the session (Fisher tests, p>0.05).

## Discussion

Our work corresponds to the first behavioural comparative investigation of human and non-human primate auditory laterality using the same experimental protocol and analyses, since authors usually compared their data on monkeys' auditory laterality with human literature (e.g. [25]). Moreover, it was the first time anybody looked at the laterality of the response to cross-categorical stimuli differing in emotional (positive and negative) and social (intra- and inter-social groups) values. We found notable differences between the two species according to the modality of responses, i.e. the monkeys frequently turned their head towards the acoustic source while the girls often just moved their gaze laterally when hearing a sound. Moreover, we evidenced auditory laterality only for intra-group negative stimuli in both species (i.e. Campbell's monkey threat calls and aggressive speech from classroom mates) but in opposite directions (respectively right and left hemisphere processing in monkeys and humans). Thus, this study suggests that auditory laterality in primates depends on both the social and emotional values of sounds heard but also that inter-species differences can be found.

We hereby discuss these results regarding 1) particularities concerning the modality of response of each species 2) the influence of the sound social value (species-specificity, degree of familiarity or affiliation) 3) the influence of the sound emotional value 4) the pertinence of behavioural responses (eye and head orientation) as visible clues to assess auditory laterality in vertebrates.

The difference in the modality of response in the two species raises interesting hypotheses on a comparative psychological perspective. Campbell's monkeys rarely just moved their eyes when hearing a sound but rather turned their head (in 357 out of 364 cases), whereas in girls proportions of eye movement and head movement were more or less equivalent (head orientations, in 121 over 208 cases). We can hypothesize that monkeys are naturally highly reactive since vigilance is crucial for their survival, whereas it is probably not as disturbing for girls to hear another girl coming from behind due to the fact that it is a common event with far less drastic consequences. Moreover, two hypotheses can explain the observations concerning monkeys. Firstly, hearing another species coming from behind is a stressful and unusual event notably given the fact that the two other species (De Brazza monkeys and red-capped mangabeys) are bigger in size. Secondly, hearing a high-ranking group-member coming from behind is also a potential source of agonism especially since the tested subject was, during the experiment, eating a rare food item. The more discrete responses of schoolgirls could be interpreted as a form of inhibition due to the presence of the experimenter facing the subject. Lee & Wagner [59] showed that adult women were more expressive when exposing their emotions alone, compared to in a context of face to face interview. For both species a high reactivity (gaze and/or head orientation) was found, supporting the pertinence of our experimental design. Post-test interviews of our schoolgirls revealed that they effectively believed that somebody was present behind the curtain (where the loudspeaker was).

The two behavioural analyses, i.e. gaze (GO) and head (HO) orientation, already used by the scientific community in the past, particularly in newborns for the discrimination of sounds, have never been combined to study auditory laterality. In our study, the GO results highlighted that the monkeys, and girls alike, oriented their gaze to the right side when hearing biological sounds. We demonstrated thus the existence of an auditory laterality in both species but however linked neither to the social or emotional values of communicative sounds. Yet, for the monkeys weak levels of gaze orientation reactivity prevented us from analysing this category of response in detail. But we confirmed the pertinence of using this GO variable in human studies. But, when taking into account either just head orientation or the first (head or eye) response, we evidenced auditory laterality socially and emotionally influenced. Interestingly our results using this paradigm highlight a right ear preference (LH) for the human subjects and therefore perfectly in accordance with previous literature. Our data show the pertinence of this non-invasive experimental protocol even though it has been highly controversed on both conceptual and empirical grounds [36], [60]. However, we also highlighted the importance of being vigilant by taking into account the nature of the behavioural variables used to measure laterality.

Secondly, our study enabled us to confirm the influence of a sound's social value on primate auditory laterality, but also highlighted differences between species. A left hemisphere specialization (LH) was found in response to conspecific speech in humans, as it has already been shown in various other species of vertebrates (Japanese macaque (*M. fuscata*): [14]; mouse (*Mus musculus*): [62], [63]; rhesus macaque (*M. mulatta*): [3], [16]; California sea lion (*Z. californianus*):[17]; mouse lemur (*M. murinus*): [33]; dog (*C. lupus*): [18]). Furthermore, our results, showing a right ear preference in humans, are perfectly in accordance with the literature (clinical, neuro-imaging, dichotic tests) expressing a left hemisphere specialization for the processing of language (cf. review in introduction). However, our female Campbell's monkeys showed a left ear preference (RH) for the processing of conspecific calls, supporting the data from several other species of vertebrates (birds of prey (*Falco tinnunculus*, *Falco subbuteo*, *Asio otus*): [36]; vervet monkey (*C. aethiops*): [25]; starling (*S. vulgaris*): [19], [20]; chimpanzee (*P. troglodytes*): [21]). From an evolutionary point of view, it is particularly interesting to see how macaques, Campbell's monkeys and vervet monkeys, belonging to different genera (respectively *Macaca*, *Cercopithecus*, *Chlorocebus*) of the same *Cercopithecinae* sub-family, expressed auditory laterality in different directions for the processing of conspecific calls, with a similar pattern for the two closer phylogenetically related species, e.g. vervets and Campbell's monkeys. More species comparisons are new needed to raise evolutionary scenario about the evolution of vocal communication and the origin of language.

Interestingly, we evidenced the existence of this auditory laterality only for sounds produced by the same group members, both in humans and monkeys, while the extra-group stimuli elicited no such asymmetry (monkeys: other species individual/humans: other classroom members). While it is clear in humans that the factor involved here is the degree of familiarity, our results in monkeys show that the emitter's specificity as well as the emitter-receiver's degree of familiarity could influence the auditory laterality. However, Petkov et al. [64] recently evidenced in rhesus monkeys a cerebral area specialized in the processing of conspecific familiar signals, an area also present in the human brain (Superior Temporal Sulcus). Some other species also exhibit an auditory laterality for the processing of familiar conspecific signals (zebra finche (*T. guttata*): [22]; bonobo (*P. paniscus*): [23]; horse (*E. caballus*): [24]).

Nevertheless, when considering only one of our two playback sessions, we found that our monkeys also gave lateralized responses to one of the four heterospecific stimuli, orienting their head to the left side after the playbacks of mangabey's threat calls, but showed no asymmetry for De Brazza monkeys. In the wild, Campbell's monkeys, like many other guenons, form polyspecific associations, often sharing trees not only with other guenons, but also with collobus and mangabeys [65], [66]. De Brazza monkeys are a guenon species that never associates itself with other monkeys. It is thus possible that Campbell's monkeys present a higher degree of social affinity with mangabeys than with De Brazza monkeys, which would support the influence of familiarity rather than specificity on auditory laterality.

Thirdly, our study permitted to confirm the influence of a sound's emotional value on primate auditory laterality. We evidenced the existence of an asymmetry for intra-group negative stimuli in humans and monkeys, and for one of the extra-group negative stimuli (EG2, mangabey's threat calls during the second session) in monkeys. Although our results suggest a differential processing according to the emotional valence of the stimuli, the directional preferences observed here were not in accordance with the Valence Theory for schoolgirls, since the girls oriented their head to the right when exposed to negative sounds. However, results concerning the monkeys complied partially with this theory, since negative calls were processed by the right side of the brain, while no lateralisation was obtained with the positive stimuli. However, the results on monkeys are perfectly in concordance with the Right Hemisphere Theory, promoting a preferential implication of the right hemisphere for the processing of highly emotional stimuli. De Boyer Des Roches et al. [67] showed a specialized processing by the right hemisphere for negative visual stimuli in horses, while no asymmetry was evidenced for positive objects. Moreover, Siniscalchi et al. [18] evidenced a specialized processing by the right hemisphere for thunderstorm auditory stimuli and highly negative conspecific calls in dogs, while conspecific calls were usually processed asymmetrically by the left hemisphere. These data could lead us to suppose that Campbell's monkeys expressed a right hemisphere specialization when exposed to highly negative calls. Our study then revealed that social and emotional factors are in fact intermingled. It suggests that the negative value of the sound heard is probably not just due to the call social function (contact *vs* threat calls in monkeys) or intonation (human voice) but also determined by the social status of the caller/speaker. Being threatened by an affiliate individual might be interpreted as particularly highly negative. Thus only intra-group, presenting a more affiliative social status than extra-group negative sounds, elicited asymmetric behaviour in monkeys and humans. Positive stimuli did not lead to significant behavioural asymmetry but positive and negative stimuli triggered significant opposite directions both in humans and monkeys. This suggests that auditory laterality must be seen as a task sharing system where both hemispheres play a role.

In conclusion, while these results could not totally confirm either the Valence or Right Hemisphere theories for both species, our data support an influence subtly balanced of the emotional and social values of sounds on human and non-human primates' auditory laterality and a differential role played by both hemispheres. Finally, this work showed the importance to take into account the nature of the behavioural variable measured and offers new perspectives for the investigating of auditory laterality, in a comparative way.

## Materials and Methods

### Subjects

We tested 7 adult female Campbell's monkey subjects, housed at the “station biologique de Paimpont” (University of Rennes I, France) in a large outdoor (29 m×9.80 m×4.20 m) - indoor (9.60 m×1.65 m×3.25 m) enclosure. The studied group matches the species' typical harem social structure [65], composed of 12 individuals: one adult male, seven adult females and offspring. Females ranged from 5 to 14 years old (11.0±1.13; mean years±s.e.). Water was provided *ad libitum*. Fruit, vegetables and monkey chows were provided twice a day after tests.

We tested thirteen schoolgirls from two different classrooms, ranging from 8 to 9 years old (8.31±0.13; mean years±s.e.), studying in the same elementary school “Duchesse Anne” in Rennes city (France).

### Stimuli selection

The vocalizations of 7 adult female monkeys from 3 different species all housed at the “station biologique de Paimpont” were recorded outdoors between March and July 2006. The stimuli individuals consisted of Campbell's monkeys (n = 3) from the same group as the subjects and of two other species living in neighbouring enclosures (n = 4). Subjects were therefore individually familiar to these seven monkeys, sharing permanent inter- or intra-group auditory and frequent visual contacts. Thus, the “extra-group stimuli - EG” were heterospecific calls, consisting of vocalisations from 2 adult female De Brazza monkeys (*Cercopithecus neglectus*) and 2 adult female red-capped mangabeys (*Cercocebus torquatus*). The “intra-group stimuli - IG” were conspecific calls, i.e. vocalizations from the 3 higher-ranked females. Having calls from three conspecific females enabled us to broadcast the call of a high-ranked female to all our subjects (since the conspecific stimuli-individuals were also used as subjects). This group had been observed on a regular basis and the hierarchy was well known (e.g. [68]–[70]). Stimuli consisted of contact calls and threat calls from each female, as defined in earlier studies (Campbell's monkeys: [57], [58], De Brazza monkeys: [71], [72]; Red-capped mangabeys: [73]). Contact calls were recorded when uttered during affiliative interactions, in a non-agonistic and non-alimentary context, and were therefore attributed a positive emotional value (P). Threat calls were recorded at the time of agonistic encounters and were therefore attributed a negative emotional value (N).

The voices of 4 girls, familiar but not family-related to the subjects were recorded. We selected schoolgirls from another classroom in the same school (extra-group stimuli – EG, n = 2, 9 years old) and from the same classroom (intra-group stimuli – IG, n = 2, 8 years old) as subjects. For EG stimuli, we selected familiar girls but not friends (evaluated by questionnaires proposed to children). For IG stimuli, we selected the most popular non-subject classroom-mates (evaluated by questionnaires proposed to children). EG and IG stimuli consisted of the same sentence, presenting a neutral emotional semantic content (“*tu fais quoi dans cette pièce*?” i.e. “what are you doing in this room?”), pronounced either with a friendly or an aggressive intonation. The positive (P) *vs* negative (N) emotional content of the stimuli was confirmed by diffusing the stimuli to 28 naïve schoolgirls of 8–9 years old and analyzing their emotional interpretation (24 item questionnaire; Wilcoxon test, n = 28, T = 2 p = 0.0001).

### Stimuli recording and preparation

Monkey calls and human voices were recorded using the same apparatus, i.e. directional microphone Sennheiser Me80 connected to a digital recorder Tascam DA-P1. They were digitized with a 44.1 kHz sample rate and a 16-bit sample size. Samples were selected when presenting a high acoustic quality (e.g. limited background noise and echoes). The sounds' amplitude was subsequently adjusted and homogenised using ANA software [74] in order to reach a loudness of 60 db SPL at the subject's ear location (measures done with Phonic Audio Analyser PAA3). This threshold was chosen in order to match the natural sounding that a subject would have received from an individual placed at the loudspeaker location.

We selected two different exemplars (calls or sentences) per monkey/child used as stimuli and per emotional category. Thus each monkey subject heard 24 strictly distinct biological sounds (2 call exemplars ×2 stimuli individuals ×2 emotions [P-contact, N-threat calls] ×3 species [IG-Campbell's monkeys, EG1-De Brazza monkeys, EG2-Red-capped mangabeys]). The girls heard 16 strictly distinct biological sounds (2 sentence exemplars ×2 stimuli individuals ×2 emotions [P-friendly, N-aggressive intonation] ×2 classrooms [IG-same, EG-different]). For the monkeys, we also broadcast a supplementary sound (pink noise, variant of a white noise) twice. The overall 26 stimuli were replicated once entirely so a total of 52 sounds were heard. Pink noises were used to control that biological sounds were behaviourally relevant to the monkeys, something we easily confirmed with the girls while interviewing them after all the tests were done. The replication of all of the tests on monkeys was done because pilot studies on auditory laterality have revealed an effect of habituation on patterns of laterality in that species (Blois-Heulin, in prep.).

### Experimental procedure

Four months were needed to familiarize the monkeys with the experimental room and with the human experimenter (M.B.) before the testing period (August 2006–January 2007). Girls were tested from April to June 2007. They had been familiarised with the experimental room, adjacent to their classroom for at least seven months and the experimenter (M.B.) spent a minimum of 2.5 days at the school with them before starting the tests. Familiarisation of the monkeys consisted in progressive isolation from the other group members (visual, auditory, both) and then a progressive habituation to voluntarily enter the experimental room and sit in the test area.

Test sessions occurred from 09.00 am to 02.00 pm for the monkeys and from 12.00 pm to 02:00 pm or 03:00 pm to 4:00 pm for the girls. Stimuli were played in a random order to prevent habituation and with a frequency of 2 to 6 trials per day per subject.

Subjects were tested individually in acoustically-homogeneous experimental rooms, that visually isolated them from their social group in order to avoid environmental and social disturbances, (i.e. monkeys: a cage built in an extension of their indoor enclosure, girls: an isolated room inside the school). Nevertheless, two female monkeys were tested while carrying their newborn baby. Subjects walked freely up to the test area facing the experimenter and were occupied with a concurrent activity (i.e. licking honey on the wire mesh, sitting on a branch for monkeys/drawing, sitting on a chair for schoolgirls). This activity, lasting a minimum of 10 seconds, was used to focus the subject's attention on something else than the experimental design, and to control the spatial position and posture of the subject during the test. Furthermore, several authors hypothesized that laterality increased efficiency of the subject to process two simultaneous tasks, e.g. foraging & vigilance [75]–[77]. Stimuli were diffused by a loudspeaker placed behind the subject (monkeys: Nagra III Kudelski mounted on the wall/girls: Sony SRS-77G, hidden behind a curtain), as soon as the subject displayed the required posture (i.e. back straight, head symmetrically positioned on the loudspeaker axis with both ears at the same distance from the loudspeaker). Stimuli were played from a Dell latitude D810 computer using Windows Media Player software. Behavioural responses were video-taped with a frontal camera for the two species. An overhead camera was also placed for the monkeys since they sometimes directed their gaze up. Observations of spontaneous behaviour within the social group and/or of randomly selected control videos (during mock experiments with no sound diffusion) revealed that subjects (1) presented no motor or sensory problems concerning head orientation and eye movement, (2) displayed no natural side-preference for head orientation (left *vs.* right).

### Analysis of behavioural responses

Videos were analysed using The Observer 5 software. We quantified three types of variables: GO – Gaze Orientation focusing only on eye movement, HO – Head Orientation, FR – First Reaction focusing on the first type of behaviour occurring, either head or gaze orientation. Any head movement considered as a way of placing one ear, rather than the other, closer to the loudspeaker was recorded as HO. Only lateral eye movements were recorded as GO. In both cases responses were considered as valid regardless of movement amplitude. We measured the latency (seconds) and the direction (left/right) for all three types of variables. We selected a reaction as a valid response when it was the first orientation (GO, HO or FR) consecutive to the sound diffusion, and when it occurred within a threshold delay of 1.0 s (GO), 1.4 s (HO) and 1.4 s (FR) for the monkeys and 1.2 s (GO), 1.4 s (HO) and 1.2 s (FR) for the girls. These threshold latencies of response were determined after visual examination of the frequency distributions of all latencies of each first movement.

### Statistical analyses

Statistical tests were performed for each variable (GO, HO and FR) on control sounds (CS) and biological sounds differing socially and emotionally, taking into account all possible combinations: intra-group positive (IGP), intra-group negative (IGN), extra-group positive (EGP), extra-group negative (EGN). In sum, four categories of stimuli were tested on the girls (IGP/IGN/EGP/EGN), whereas a total of seven categories were tested on the monkeys, since they heard calls from two extra- groups and a pink noise (IGP/IGN/EG1P/EG2P/EG1N/EG2N/CS). Firstly, Wilcoxon signed rank tests were applied to each category mentioned above in order to test reactivity (presence *versus* absence of response) and the direction of laterality (left *versus* right preference). Then Fisher tests were done to compare the patterns of laterality (1) between the different emotional values of the sounds within each social category (IGP *vs* IGN, EGP *vs* EGN, EG1P *vs* EG1N and EG2P *vs* EG2N) and (2) between the different social values within each emotional category (e.g. IGP *vs* EGP, IGN *vs* EGN, IGP *vs* EG1P…). At last, only for the monkeys, were analyses performed for the whole experiment (total session, STOT), as well as for a first (S1) and second session (S2).
